# The Journey of Arsenic from Soil to Grain in Rice

**DOI:** 10.3389/fpls.2017.01007

**Published:** 2017-06-20

**Authors:** Surabhi Awasthi, Reshu Chauhan, Sudhakar Srivastava, Rudra D. Tripathi

**Affiliations:** ^1^CSIR-National Botanical Research Institute, Council of Scientific and Industrial ResearchLucknow, India; ^2^Institute of Environment and Sustanaible Development, Banaras Hindu UniversityVaranasi, India

**Keywords:** Arsenic, iron plaque, phytochelatins, sulfur, transporters

## Abstract

Arsenic (As) is a non-essential toxic metalloid whose elevated concentration in rice grains is a serious issue both for rice yield and quality, and for human health. The rice-As interactions, hence, have been studied extensively in past few decades. A deep understanding of factors influencing As uptake and transport from soil to grains can be helpful to tackle this issue so as to minimize grain As levels. As uptake at the root surface by rice plants depends on factors like iron plaque and radial oxygen loss. There is involvement of a number of transporters *viz*., phosphate transporters and aquaglyceroporins in the uptake and transport of different As species and in the movement to subcellular compartments. These processes are also affected by sulfur availability and consequently on the level of thiol (-SH)-containing As binding peptides viz., glutathione (GSH) and phytochelatins (PCs). Further, the role of phloem in As movement to the grains is also suggested. This review presents a detailed map of journey of As from soil to the grains. The implications for the utilization of available knowledge in minimizing As in rice grains are presented.

## Introduction

Arsenic (As) is a naturally occurring toxic metalloid that occurs in many minerals, often in combination with sulfur. The problem of As contamination is widespread throughout the world including parts of United States, China, Europe and Southeast Asia. However, the extent of As contamination is most severe in Southeast Asia including Bangladesh and some states of India (Srivastava et al., 2016b). The primary sources of As are thought to be eroding coal seam and rocks containing sulfide minerals within the Himalayas whose weathering and transport leads to downstream deposition of As in Gangetic plains (Acharya et al., 1999). The minerals contained within these deposits are oxidized when exposed to the atmosphere, and much of their As content is transferred to secondary phases including iron (Fe) hydroxides, oxyhydroxides, and oxides, collectively referred to as Fe oxides. As is released from Fe oxides into groundwater through microbial processes (Fendorf et al., 2010). Hence, As has been found to be distributed in widespread areas of Gangetic plains spread across Uttar Pradesh, Bihar and West Bengal. Large areas of paddy soils are contaminated by As due to irrigation with As-tainted groundwater. Drinking water and crops (due to transfer of As in the food chain via groundwater-soil-plant system) are the major sources of As to humans. As and its compounds are used for the production of pesticides, herbicides and insecticides; nonetheless these applications are declining. Only a few bacterial species thrive on As since they employ it in their respiratory metabolism (Yang and Rosen, 2016). As is a highly toxic metalloid to plants, animals and humans. In humans, other than cancers, As has been found to be associated with several health problems, including genotoxic effects (Banerjee et al., 2013). The permissible limit of As in drinking water is 10 μg/L as per WHO guidelines (World Health Organization [WHO], 2004). However, in many developing countries including Bangladesh, 50 μg/L is the commonly adopted guideline. As contamination harms physiochemical properties of soils and leads to loss of crop yields (Miteva, 2002; Rahman et al., 2007).

**FIGURE 1:**
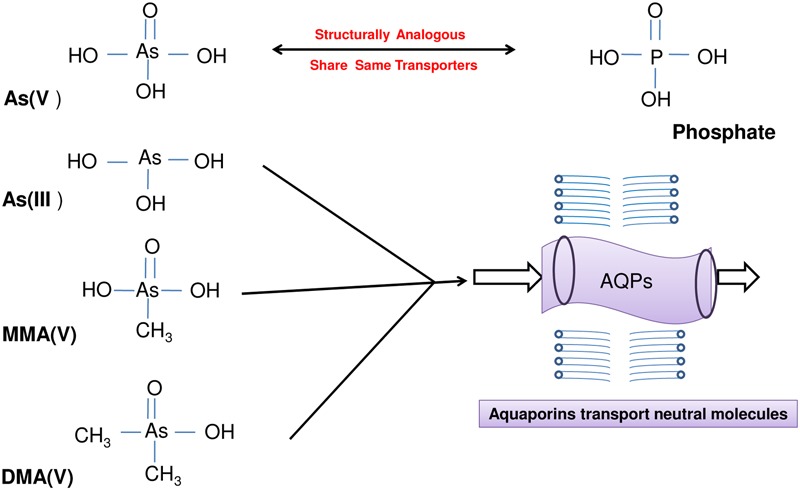
A diagrammatic presentation of different inorganic and organic arsenic species present in the environment. The uptake and transport of arsenate [As(V)] occurs through phosphate transporters due to structural analogy while that of arsenite [As(III)], monomethylarsonic acid [MMA(V)] and dimethylarsinic acid [DMA(V)] via aquaglyceroporins transporting neutral molecules.

Arsenic exists in the environment in several inorganic and organic forms with arsenate [As(V)] and arsenite [As(III)] being the most prevalent inorganic and toxic forms of As (**Figure 1**). Arsenate being a phosphate analog interferes with phosphate metabolism (phosphorylation and ATP synthesis) in plants while As(III) binds to sulfhydryl groups of proteins affecting their structures and/or catalytic functions (Tripathi et al., 2007; Zhao et al., 2010). Several studies report the increased production of reactive oxygen species (ROS) during As stress that leads to membrane damage, non-specific oxidation of proteins and membrane lipids and also causes DNA injury (Pastori and Foyer, 2002; Apel and Hirt, 2004; Srivastava et al., 2011). To cope with As stress-induced oxidative stress, plants have evolved ROS-scavenging enzymatic and non-enzymatic antioxidants (Apel and Hirt, 2004; Chauhan et al., 2017). In addition, sulfur metabolism plays crucial role in tackling As inside the plants as sulfhydryl (-SH) containing short peptides like glutathione (GSH) and phytochelatins (PCs) bind As and help in its sequestration to vacuoles (Batista et al., 2014). An increase in the production of PCs upon exposure to As in rice has been observed (Srivastava et al., 2016a). In addition, a coordination of various metabolic pathways viz., carbon, nitrogen and sulfur metabolism is essential to tolerate As stress effectively (Pathare et al., 2013). Through a comparative analysis between tolerant and sensitive variety of *Brassica juncea*, Pathare et al. (2013) proposed that γ-aminobutyric acid (GABA) may act as a central metabolite in regulation of coordinated response of carbon, nitrogen and sulfur metabolism under As stress so as to increase production of GSH and PCs without affecting normal cellular functioning. Further, the tolerant variety of plants like *B. juncea* has mechanisms in place to sense the As stress at a very early phase so as to tolerate it effectively; while a sensitive variety lacks such a coordinated mode of action. Such a sensing has been proposed to rely on sensing the sulfur status of plants as an indirect perception of As stress (Srivastava and D’souza, 2009). The signaling mechanisms involve the participation of several players viz., phytohormones (jasmonates, auxins, cytokinins, ethylene, etc.), kinases, transcription factors, microRNAs, ROS and nitric oxide (NO) (Leterrier et al., 2012; Rao et al., 2012; Yu et al., 2012; Srivastava et al., 2013, 2015). MicroRNA528 has been identified to be specifically involved in the regulation of As(III) tolerance in rice (Liu et al., 2015).

Rice is one of the most severely affected crop plants with As contamination as compared to other crop plants like wheat and maize. The reason being the cultivation method of rice that is flooded as compared to non-flooded for wheat. This leads to the development of reducing conditions in soils that in turn result in predominance of As(III) over As(V). Further, rice is one of the most efficient silica accumulators among all crop plants and As(III) too enters through silicic acid transporters in rice (Ma et al., 2008; Norton et al., 2010). These factors contribute to As accumulation in rice grains in quantities greater than recommended safe limits (Zhao et al., 2010). The situation becomes of even grave concern considering the very high rice consumption rate in As contaminated South east Asian countries ranging from 250 to 650 g of rice per day per person (Arslan et al., 2017). It is imperative to understand the mechanisms of As uptake and translocation by rice (Li et al., 2011). The present review focuses on the journey of As from soil to rice grains.

## Factors Affecting Arsenic Availability and Speciation at Root Level: Iron Plaque and Radial Oxygen Loss

**FIGURE 2:**
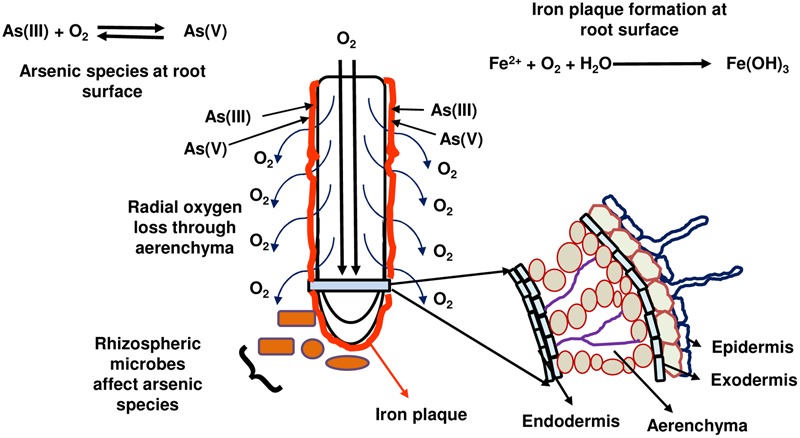
The influence of radial oxygen loss and iron plaque formation on rice root surface on arsenic species availability to rice and consequently arsenic uptake by roots. Rice roots release oxygen through aerenchyma (radial oxygen loss) into soil that oxidizes ferrous ion to ferric ion forming precipitate (iron plaque) at root surface. Oxygen also oxidizes arsenite to arsenate that gets adsorbed on to iron plaque. However, rhizospheric microbes also play a role in inter-conversion of arsenic species.

Rice is a semi-aquatic plant and, similar to wetland plants, has extensive aerenchyma in its roots. This aerenchyma allows O_2_ infiltration from shoots for respiration activity in roots. To cope with the anaerobic conditions in submerged soil, aerenchyma of rice roots releases a part of the O_2_ to the rhizosphere. This is referred to as radial oxygen loss (ROL) and varies from genotype to genotype and depends on the waterlogging and/or O_2_ availability in soils (Colmer et al., 2006; **Figure 2**). Owing the O_2_ release, ferrous iron (Fe^2+^) gets oxidized to ferric iron (Fe^3+^) and results in formation of precipitate of iron oxides/hydroxides on the root surface. This is called as iron plaque, which has the characteristic orange color (**Figure 2**). The oxides and hydroxides of Fe are strong sorbents for As and hence iron plaque becomes a major sink of As containing even greater amounts of As than that in roots (Liu et al., 2006). Further, chemical speciation of As in iron plaque has also been done by X-ray absorption near-edge structure (XANES) and it has been found to be mainly As(V) (Liu et al., 2006; Seyfferth et al., 2010; Frommer et al., 2011). As has also been found in iron plaque present in apoplast of root epidermal cells (Moore et al., 2011). However, the contribution of iron plaque in As concentrations in rice plants is debatable. It is found to act as barrier to As(V) entry in some studies (Liu et al., 2004) while a source of As(V) in others (Liu et al., 2006). Further, different roles of iron plaque as a barrier or source of As have been found for different As species viz., As(V) and As(III) (Chen et al., 2005). The issue of iron plaque has been discussed in depth in a recent review by Tripathi et al. (2014). A role of iron plaque in influencing As concentrations may also vary as per the level of iron plaque on root surface that indeed varies from root tips to old roots and from primary to lateral roots (Seyfferth et al., 2010; Frommer et al., 2011). In pot studies conducted by Mei et al. (2009) and Wu C. et al. (2011) with 20–25 cultivars, As in rice grains was found to be negatively correlated to root porosity and ROL from roots of the cultivar. Thus, a cultivar with more oxygen release from roots allowed greater iron plaque formation to reduce As uptake. Further, more oxygen release may also oxidize As(III) to As(V), which is more strongly adsorbed to iron plaque. Dwivedi et al. (2010) observed significant positive correlation in iron plaque on rice roots in field trials with that of Fe and As with up to 75–89% As concentrated in iron plaque. Meng et al. (2002) observed that Fe hydroxides show strong binding affinity to As(V) and reduce As translocation to shoots. The property of iron plaque formation has been found to vary owing to genotypic variations of rice cultivars during field trials in the West Bengal, India (Dwivedi et al., 2010). An important determinant of As species present on iron plaque has been recently found to be the microbial composition. Contrasting differences were found in the microbial composition and diversity between iron plaque and, bulk and rhizosphere soils. Further, there were As(III)-oxidizing bacteria present on root iron plaque (viz., *Acidovorax* and *Hydrogenophaga*) that were involved in As transformation and hence influence As concentration in rice tissues (Hu et al., 2015). Silica also influences the formation of iron plaque on rice roots and hence controls the As concentration in iron plaque as well as in rice plant. But, it also varies from genotype to genotype and ROL abilities (Wu et al., 2015, 2016). It is important to consider that As itself can influence ROL and iron plaque formation on rice roots (Wu et al., 2013).

## Transporters for Uptake and Translocation of Arsenic

Plants acquire essential and beneficial elements from the soil through transporters. But, the selectivity of transporters is imperfect and they can also take up nonessential elements. In inorganic forms of As, As(III) is more toxic than As(V) and they differ in their mode of toxicity as well as their transport in plants. Arsenate finds its way into plants through phosphate transporters (PHTs) (**Figure 1**). Till date a number of phosphate transporters have been identified for the As(V) uptake in different plants. Arsenate enters into the cell via OsPHT1;1 (Kamiya et al., 2013), OsPHT1;8 (Wu Z. et al. 2011) in rice and AtPHT1;1, AtPHT1;4, AtPHT1;5, AtPHT1;7, AtPHT1;8, AtPHT1;9 in *A. thaliana* (Shin et al., 2004; Catarecha et al., 2007; Remy et al., 2012; LeBlanc et al., 2013; Fontenot et al., 2015). Wang et al. (2016) compared As(V) tolerance of *aus* variety Kasalath with *japonica* variety Nipponbare and found Kasalath to be more tolerant to As(V) than Nipponbare. This could be attributed to 2- to 3-fold higher expression of *OsPT2* and *OsPT8.* The *ospt8* mutants of both Kasalath and Nipponbare had a reduction of 33–57% in As(V) uptake and showed increased tolerance to As(V). Thus, OsPT8 was identified as an important transporter for As(V) uptake in rice. The root-to-shoot As(V) transport also occurs through different PHT proteins (Catarecha et al., 2007; Zhao et al., 2010; Mendoza-Cózatl et al., 2011; Wu Z. et al. 2011). The regulators of phosphate transport viz., OsPHF1 (phosphate transporter traffic facilitator 1) and PHR2 (phosphate starvation response 2) also have an effect on As(V) uptake and transport (Wu Z. et al., 2011). In contrast, As(III) and undissociated methylated As species are transported through aquaglyceroporins of various classes and more predominantly via nodulin 26-like intrinsic protein (NIP) class of aquaporin channels (Zhao et al., 2010; Mosa et al., 2012; **Figure 1**). A NIP class transporter, OsNIP2; 1 (Lsi1) is well known transporter for silicic acid (Si), which has a major role in As(III) uptake (Ma et al., 2008). AtNIP1;1, AtNIP1;2, AtNIP5;1 (Kamiya and Fujiwara, 2009), AtNIP3;1 (Xu et al., 2008), AtNIP6;1 (Bienert et al., 2008), AtNIP7;1 (Isayenkov and Maathuis, 2008) facilitate As(III) uptake in *A. thaliana* and OsNIP1;1, OsNIP2;2 (OsLsi6), OsNIP3;1 (Ma et al., 2008), OsNIP3;2 (Bienert et al., 2008), OsNIP3;3 (Katsuhara et al., 2014) in rice. Recently, an important role of OsNIP3;2 in As(III) uptake by lateral roots was demonstrated in rice (Chen et al., 2017). The gene OsNIP3;2 was found to be predominantly localized in lateral roots and stele region of primary roots and its mutation led to reduced As(III) concentrations in roots but not in shoot. OsLsi2 is involved in AsIII efflux and xylem loading (Ma et al., 2008). Mosa et al. (2012) reported the involvement of aquaporins of PIP (plasma membrane intrinsic proteins) class including OsPIP2;4, OsPIP2;6, and OsPIP2;7 for As(III) uptake and transport. A role of other transporters like NRAMP1 (Natural Resistance-Associated Macrophage Protein 1) (Tiwari et al., 2014) is also suggested in As(III) uptake and transport. In a comparative study employing six contrasting genotypes of rice (three high arsenic accumulating genotypes and three low arsenic accumulating genotypes), transcriptome profiling demonstrated contrasting expression patterns in the genotypes. The profiling data included two aquaporin-coding genes (Os06g12310 and Os02g51110), which showed high expression in one low arsenic accumulating genotype, Nayanmoni while down regulation in two other low-grain genotypes (CN1646-5, CN1646-2). Another aquaporin gene (Os07g26630) was upregulated in CN1646-2 and CN1646-5 but down regulated in Nayanmoni. Hence, there may be other aquaporin gene responsible for As uptake and transport, which may show variations in expression in different genotypes (Rai et al., 2015).

Once As enters into the plants, it exists mostly in its reduced form, i.e., As(III) that may get transported to vacuoles either as such via PvACR3 (As Compounds Resistance) in *Pteris vittata* (Indriolo et al., 2010) or after complexation with phytochelatins and then as PC-As(III) complexes via the members of ABC (ATP Binding Cassette) transporter family, ABCC1 and ABCC2 in Arabidopsis (Song et al., 2010) and rice (Song et al., 2014). There is a lot yet to be revealed about transporters involved in As loading from xylem to phloem and into seeds. Very recently, progress in this direction has been made and transporters for phloem loading of As in the form of As(III) have been identified as inositol transporters (INTs) known for inositol uptake in phloem in Arabidopsis. The disruption of inositol transporters (INT2 and INT4) in Arabidopsis resulted in decreased As in phloem, silique and seeds as compared to wild-type plants (Duan et al., 2016). There is need to identify these transporters in rice also.

The uptake of monomethylarsonic acid (MMA) and dimethylarsinic acid (DMA) has also been found to occur through Lsi1. However, Lsi2 was not found to be permeable to DMA (Li et al., 2009). Inorganic and organic As species differ in their mobility. Zhao et al. (2012) performed an experiment with radioactive As (^73^As) for 2–4 days and found that out of total As(III) taken up by rice plants, only 10% reached to the shoots and only 3.3% to the grain. In contrast, the mobility of organic As species is greater than inorganic As species (Carey et al., 2010, 2011; Ye et al., 2010). This has been attributed to the phytochelatins (PCs) mediated complexation and storage of inorganic As (Raab et al., 2007; Moore et al., 2014). In the mobility of As, nodes act as a controlling point as they remain connected through their connections to both upper and lower nodes (Yamaji and Ma, 2014). Nodes regulate the As storage and its distribution to the rice grain (Yamaji and Ma, 2014; Zhao et al., 2014). Moore et al. (2014) found much higher concentration of As in the nodes than internodes and leaves. In agreement to earlier studies, Chen et al. (2015) confirmed that rice nodes limited the As(III) distribution into the grain by acting as As(III) filter. The ABCC transporter, localized in tonoplast of phloem cells in nodes, mediates PC-As(III) complex transport to vacuoles (Song et al., 2014). Knockout mutants of *osabcc1* showed higher As accumulation in grains but lower As in nodes than WT (Song et al., 2014). Since OsABCC1 is a vacuolar PC–As(III) transporter, it may sequester PC–As in vacuoles in nodes in WT but not in mutant. Moore et al. (2014) found OsABCC1 localized in the phloem companion cells of the vascular bundle in nodes strengthening that OsABCC1 inhibits the translocation of As into grains by transporting PC-As complexes into vacuoles of phloem cells in nodes.

The regulation of expression and localization of transporters is also important in As tolerance. AtPht1;1 is regulated by transcription factor WRKY6 and WRKY45 to modulate As(V) uptake (Castrillo et al., 2013; Wang et al., 2014). Mohan et al. (2016) found As(V) tolerance in Arabidopsis mutants for cytokinin signaling. Cytokinin depletion was found to activate a coordinated activation of As(V) tolerance mechanisms that included increased synthesis of PCs and GSH. Hence, cytokinin plays regulatory role in As stress tolerance. Another regulator of NIP1;1 in Arabidopsis has been found to be a calcium-dependent protein kinase (CPK31). The mutant of *cpk31* improved tolerance of Arabidopsis plants similar to *nip1;1* mutation to As(III) and the double mutant *cpk31 nip1;1* had even greater tolerance to As(III) as compared to that of *cpk31* mutant (Ji et al., 2017). Hence, regulatory elements may affect transporter expression and activity to modulate As tolerance. There is need to identify such specific regulators in rice also.

The speciation of As is an important determinant of its uptake and transport in plants. Arsenate reductase (AR) is a crucial enzyme in plants regulating the conversion of As(V) to As(III). Several AR genes have been discovered in plants though with a questionable role /contribution in As(V) reduction (Zhao et al., 2009; Chao et al., 2014). In Arabidopsis, lately two AR genes have been identified namely ATQ1 (arsenate tolerance QTL1; Sánchez-Bermejo et al., 2014) and HAC1 (High As Content1) (Chao et al., 2014). HAC1 has been found to reduce As(V) to As(III) in the outer cell layer of the root and to facilitate As(III) efflux from the roots to the soil (Chao et al., 2014). In rice also, two orthologous genes of HAC1 viz., OsHAC1;1 and OsHAC1;2 function as As(V) reductases (Shi et al., 2016). OsHAC1;1 and OsHAC1;2 both are expressed mainly in roots. However, their localization is different with OsHAC1;1 being abundant in epidermis, root hairs and pericycle while OsHAC1;2 being predominant in epidermis, outer cortex layers and endodermis. OsHAC1;1 also shows significant expression in stems and nodes (Xu et al., 2017). Xu et al. (2017) have recently identified HAC4 as an As(V) reductase from rice with expression in root elongation and maturation zone in epidermis and exodermis but no expression in leaves. The mutation of OsHAC1;1, OsHAC1;2 (Shi et al., 2016) and OsHAC4 (Xu et al., 2017) led to decrease in As(V) reduction in roots and consequently decreased As(III) efflux and increased As accumulation in shoots. In contrast, overexpression of these genes produced opposite effects.

**FIGURE 3:**
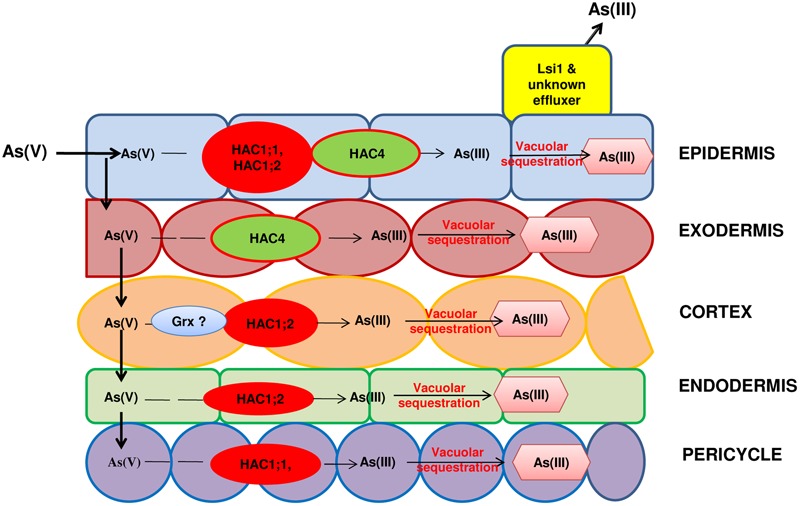
The arsenate reductases in roots are involved in reduction of As(V) to As(III) and hence, influence both efflux of As(III) back to soil or upward movement of As(III) to shoot. HAC: (High Arsenic Content) Arsenate reductases of group 1 and group 4, Grx: Glutaredoxins. Question marks indicate knowledge gaps about exact location of Grx in rice root.

Glutaredoxins (Grxs) are ubiquitous low molecular weight, cysteine-rich multifunctional proteins that take part in various cellular processes including maintenance and regulation of cellular redox state and protection under oxidative stress (Lillig et al., 2008). Recently, Verma et al. (2016a) characterized a Grx gene from rice (OsGrx) by cloning and expression of OsGrx_C7 and OsGrx_C2.1 in *Escherichia coli* and *Saccharomyces cerevisiae* mutant strains. It was found to result in increased tolerance to As(V) and As(III) presumably through increased As(V) reduction and As(III) extrusion. Over-expression of OsGrx_C7 and OsGrx_C2.1 in *Arabidopsis thaliana* conferred As tolerance and reduced As accumulation in seeds and shoot tissues compared to WT plants. Thus, OsGrx_C7 and OsGrx_C2.1 are another important determinant of As-stress response in plants (Verma et al., 2016b). Hence, As(V) reduction is an important step of As detoxification in plants both for its onward transport and also for its complexation and storage (**Figure 3**). Further, As(V) reduction influences the grain As accumulation (Shi et al., 2016).

An important role of transpiration in As translocation from root-to-shoot has been revealed in *P. vittata.* It was observed by Wan et al. (2015) that subjecting the plants to shade to reduce transpiration by 28–67% decreased shoot As by 19–56%. They further compared ecotypes of *P. vittata* from moister and warmer habitat having high transpiration rate with ecotypes from drier and cooler habitat and found that ecotypes with higher transpiration also had higher As in shoot.

## The Transport and Subcellular Distribution of Arsenic: The Influence of Sulfur

**FIGURE 4:**
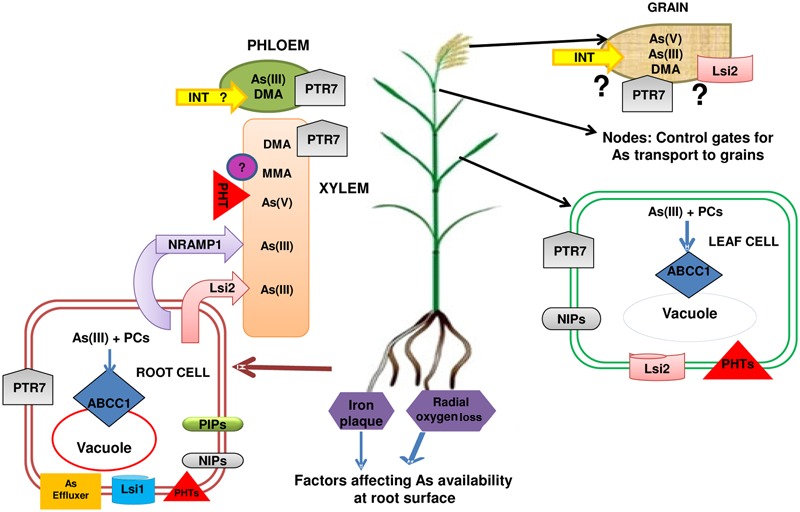
A summarized flow diagram of factors influencing arsenic availability around rice roots and transporters involved in arsenic uptake and transport through xylem and phloem up to the grains. Phosphate transporters, PHTs (OsPHT1;1, OsPHT1;8) and aquaglyceroporins [OsNIP1;1, OsNIP2;1 (Lsi1), OsNIP3;1, OsNIP2;2 (Lsi6), OsPIP2;4, OsPIP2;6, OsPIP2;7] are involved in uptake of As(V) and As(III), respectively, in rice plant. Lsi1 in root epidermis also acts as As(III) effluxer. OsNIP3;2 & OsNIP3;3 contribute in transport of As(III) in rice plant. OsLsi2 and Nramp1 (Natural resistance-associated macrophage protein) help in xylem loading of As and in root to shoot transportation. ABCC, ATP-binding cassette transporter help in vacuolar sequestration of As(III) complexed with thiolic compounds. Arsenic is transported into seeds through phloem. Inositol transporters (INTs) are known to be involved in phloem loading of As(III) in Arabidopsis and thus in the transportation of As(III) to seeds. It is hypothesized that such INTs may help in transportation of As(III) in rice grains through phloem loading. OsPTR7 (Putative Peptide transporter) is involved in long distant transportation of DMA from roots to grains. The transporter of As(V) and As(III) in grains are yet to be revealed though Lsi2 has been considered as a probable candidate. Question marks indicate knowledge gaps.

Arsenic has been found to be hindered by sulfur (S) supply during its uptake, translocation and accumulation in rice plants, however, variable results have been observed (Hu et al., 2007; Zhang et al., 2011; Dixit et al., 2015). A recent study by Srivastava et al. (2016a) evaluated the effect of varying sulfur (S) supply on As accumulation and distribution in rice (*Oryza sativa*) plants. They have found more significant decrease in As accumulation at zero S (0.003 mM) supply in comparison to normal S (0.798 mM). This was accompanied by the changes in the subcellular distribution of As. More synthesis of thiols including phytochelatins has been observed even at zero S supply. Thus, S availability is an important criterion to tackle As stress and plants continue to rely on thiol metabolism even when S supply is extremely limited. A similar response was found with increase in PC synthesis despite decline in S level in a SULT1;2 (Sulfate Transporter, Group 1) mutant of Arabidopsis. This was found to enhance As sensitivity of plants (Nishida et al., 2016). There are several reports on decreased As concentrations in shoots at high doses of S that was attributable either to S induced formation of iron plaque or to increased complexation of As in roots (Hu et al., 2007; Zhang et al., 2011; Dixit et al., 2015). Recently, high S supply mediated decline in As concentration has also been reported in rice grain (44%) in comparison to no S (0 S) supplied plants (Zhang et al., 2016). The high sulfur supply was found to regulate the expression of genes involved in As metabolism viz., down-regulation of the phosphate transporter (PT): OsPT23 and aquaporin gene: OsTIP4;2 (Tonoplast Intrinsic Protein), while upregulation of ABC transporter genes (OsABCG5, OsABCI7_2and OsABC6) and phytochelatin synthase genes (OsPCS1, OsPCS3 and OsPCS13) (Zhang et al., 2016; **Figure 4**). It suggests that high sulfur supply allowed plants to synthesize more PCs and sequestrate As to vacuoles efficiently by complexation with PCs via upregulation of PCS and ABC transporter genes. Sulfur supply also seems to reduce As(V) and As(III) uptake at roots through down regulation of phosphate transporter and aquaporin channel, respectively. It is important to note that an aquaporin channel of TIP class (TIP4;1) is suggested to be involved in As(III) uptake in *P. vittata* in a recent study by He et al. (2016). Yang et al. (2016) identified a transporter OsCLT1 [CRT (Chloroquine-Resistance Transporter)-Like transporter) in rice, which is localized to plastids. The mutant lines (Osclt1) had decreased PC2 levels as compared to WT upon exposure to As(V) or As(III) and showed a decrease in As concentration. Thus, OsCLT1 regulates PC biosynthesis by maintaining glutathione homeostasis.

## The Mobility of Arsenic Through Xylem and Phloem

The mobility of As has been found to depend on its speciation with inorganic As species having lower root to shoot mobility than organic As species. Hence, As is concentrated mostly in roots leading to low shoot/root ratios of 0.1–0.3 in rice (Zhao et al., 2009). Arsenate and As(III) share the transporters for their uptake in rice with phosphate (P) and silicic acid (Si). But, Si shows greater mobility than As with ratio of xylem sap concentration to that in external medium being around 20 for Si as compared to 0.3–0.6 for As (Mitani and Ma, 2005; Zhao et al., 2009; Meharg and Zhao, 2012). The difference in mobility might be due to complexation of As by PCs in roots that does not occur in case of P and Si. Yet, the level of As concentrations are much higher in rice than that in other cereals that has been attributed to specific localization of influx and efflux transporters of As in exodermis and endodermis cells (Su et al., 2010) allowing efficient uptake and export of As to xylem. Arsenite has been found as the predominant species in the xylem sap of rice whether supplied with As(V)/As(III) (Ma et al., 2008; Zhao et al., 2009; Su et al., 2010). However, with As(V) supply, initially As(V) was in larger proportion up to 2 h and then the concentration of As(III) gradually increased. Constitutive expression of phosphate transporter (OsPht1;8) increased the uptake and xylem loading of As(V) (Wu Z. et al., 2011). In xylem sap, no As-thiol complexes were detected in sunflower and castor bean (Raab et al., 2004; Ye et al., 2010). As suggested earlier, thiol complexation restricts As(III) movement, while for organic As species, this is not a restriction for movement. Another, factor suggested to be responsible for mobility differences is hydrophobicity of inorganic and organic As. For organic As species, only pentavalent species viz., MMA(V) and DMA(V) were detected in xylem sap (Li et al., 2009; Ye et al., 2010).

Although, few reports are available for xylem transport of As, there is scanty information about the mechanisms of phloem mediated transport of As to grains. Likewise xylem sap, As(III; 70–94%) is also a predominant form in phloem sap of castor bean (Ye et al., 2010). This As(III) is present in free form despite the presence of thiols in phloem sap owing to the fact that pH of phloem sap is high (up to 8) that makes As-thiol complexes unstable. MMA(V) and DMA(V) have also been detected in phloem sap and their levels were higher than xylem sap (Ye et al., 2010). In a study, synchrotron μX-ray fluorescence (μ-XRF) was applied to analyze As distribution in the top node and internode of a *lsi2* mutant and WT of soil grown plants and also in excised panicles (Chen et al., 2015). As was found to be stored in phloem in the top node and internode with lsi2 mutants having lower As accumulation in phloem as compared to WT. In excised panicles, lsi2 mutant distributed less As to the grain compared to WT, when supplied with As(III), whereas no difference was noticed when DMA was supplied externally. The inhibition of PC production by L-buthionine sulfoximine (BSO) led to increase in As in grains. Hence, rice nodes were designated to act as filters restricting As(III) distribution to the grain with important roles of Lsi2 and PC levels (Chen et al., 2015).

## Transport of Arsenic into Rice Grain

The nutrients are transported through the ovular vascular trace to the endosperm via chalaza to nucellar projection and finally to the endosperm (Krishnan and Dayanandan, 2003). A symplastic discontinuity exists during transport of nutrients between maternal (OVT, chalaza, nucellar projection, nucellar epidermis) to filial (endosperm, aleurone layer and embryo) tissues that may act as a bottleneck step for mineral nutrient transport. As transportation also follows the same route. Carey et al. (2010) analyzed inorganic As and DMA transport to developing rice caryopsis by feeding cut rice panicles with As(III) or DMA. The transport of DMA to rice grains was more efficient despite the concentration of DMA (13.3 μM) in the feeding solution being only one-tenth of that of As(III; 133 μM), rice grain accumulated higher As (17-fold) from the DMA treatment. Stem girdling that removed phloem decreased the grain As by 55% and 90% in the DMA and As(III) treatments, respectively. This suggested that phloem is the primary route of transport to grains for As(III), while for DMA, phloem and xylem are equally important. In agreement to this, Carey et al. (2011) also reported that inorganic As is poorly transported to grain through phloem transport, while organic species [DMA(V) and MMA(V)] are transported very efficiently. Moreover, it was also hypothesized that stem translocation of inorganic As may not rely solely on Si transporters (Carey et al., 2011). Zhao et al., (2012) also found almost complete blockage of 73As entry into rice grains (97% decline) when stem girdling was done in rice. The differences in mobility of inorganic and organic As are also visible in their distribution pattern as inorganic As is present in OVT region (Meharg et al., 2008; Lombi et al., 2009) while DMA has been found in the endosperm of rice grain (Moore et al., 2010; Norton et al., 2010; Zheng et al., 2011). In brown rice, bran As is mainly inorganic, while endosperm contains mostly DMA (Sun et al., 2008). Very recently, a putative peptide transporter from rice [OsPTR7 (OsNPF8.1) peptide transporter] has been characterized by Tang et al. (2017). This transporter shows significant expression during grain filling stage in roots, leaves and node I. The mutants of OsPTR7 had no detectable DMA in grains in field conditions compared to 35% As in the form of DMA in WT plants. Hence, OsPTR7 is a long distance transporter for root to shoot translocation and grain transport of DMA (**Figure 4**). As mentioned above, INT2 and INT4 in Arabidopsis also function in regulation of As accumulation in grains (Duan et al., 2016).

In addition to this, genotypic and environmental factors also influence the rice plant As profile in terms of As concentrations, speciation and distribution. Norton et al. (2009) conducted an experiment with 13 common cultivars at six sites of three countries (Bangladesh, India and China) and found that largest factor responsible for grain As variation was environment followed by genotype and genotype-environment interaction. Similarly, Ahmed et al. (2011) also observed large influence of environment, genotype and genotype-environment on grain As variation when compared the grain As levels in 38 cultivars grown at 10 sites in Bangladesh. Pillai et al. (2010) also found significant variation in grain As and As speciation in 25 cultivars grown in United States with major contributing element being the genotype and also found significant positive correlation between grain As(III) and DMA concentrations in a time dependent manner. Thus, the time of vegetative growth period has an impact on grain As levels. Syu et al. (2015) estimated the As content and species in six rice genotype in Taiwan and found that As content in grains were either equal or higher in Indica genotypes than that in Japonica genotypes. Dave et al. (2013) conducted laboratory based screening of 303 rice genotypes to arsenite exposure and found significant variation in tolerance of genotypes along with a maximum of 13-fold difference in As accumulation between tolerant (IC-340072) and sensitive (IC-115730) genotypes. Dwivedi et al. (2012) cultivated 90 rice germplasms in Chinsurah and reported significant variation in grain total As accumulation among germplasms. Hence, identifying genotypic variation is an important step toward development of safe rice cultivar for cultivation in As affected areas. Indeed, on the basis of continued efforts to characterize low grain arsenic accumulating genotypes through experiments both at laboratory and field level, a potential genotype (CN-1794-2-CSIR-NBRI) has been utilized for the development of a variety named Muktashri jointly by Rice Research Station, Chinsurah and CSIR-National Botanical Research Institute, Lucknow, which has very low arsenic accumulation in grains with high yields (As of July 31, 2015, Rice outlook gave news of release of low arsenic rice variety).

## Strategies for Safe Rice

The knowledge gained over the years with respect to As uptake, transport and sequestration, though incomplete, opens up possibilities for ensuring safety of rice and reducing As level in its grains. The important factors eventually leading to effects on As level in grains include factors regulating As concentration and speciation in soil, transporters involved in the journey of As from soil to grain and chelators / tissues influencing mobility of As. The attempts have been made to reduce As concentration in rice grains through different approaches.

(a) Agronomic practices to regulate the availability of As to rice plants: The notable examples of agronomic practices include water management (Spanu et al., 2012; Moreno-Jiménez et al., 2014), fertilizer amendments (Seyfferth et al., 2016) and mycorrhizal (Poonam et al., 2017) and microbial (Lakshmanan et al., 2016) treatments. However, these approaches either yielded contrasting results or increased the concentration of other toxic element like Cd while decreasing that of As (Moreno-Jiménez et al., 2014; Poonam et al., 2017).
(b) Altering the expression of transporters involved in uptake, transport and sequestration of As: Transporters of As(V) and As(III) uptake (Wu Z. et al., 2011; Mosa et al., 2012) and vacuolar sequestration (Song et al., 2014) have been targeted leading to decrease in As accumulation in rice shoot and grains. However, altered expression of transporters of As, which are essentially the transporters of essential elements like phosphate, silica, boron and compounds like water, is questionable considering possible influences on homeostasis of essential elements and compounds (Tripathi et al., 2007). The increased vacuolar sequestration needs to be studied in light of influence on homeostasis of other essential elements like Zn, which can also be complexed by PCs (Tennstedt et al., 2009). The transgenics hence need to be analyzed for multiple nutrient elements.
(c) Targeting the mobility of As through enhancing the synthesis of chelators and through changes in its speciation: This approach aimed to enhance GSH and PC synthesis (Tripathi et al., 2007) and to enhance methylation and volatilization of As (Meng et al., 2011). The increase in the synthesis of GSH and PCs has been found to yield contrasting results owing to stress exerted to sulfur metabolism (Tripathi et al., 2007). The approach of methylation and volatilization of As into the atmosphere to reduce As in rice grains is debatable as this may be toxic to farmers working in the field and local people residing in the area.

**FIGURE 5:**
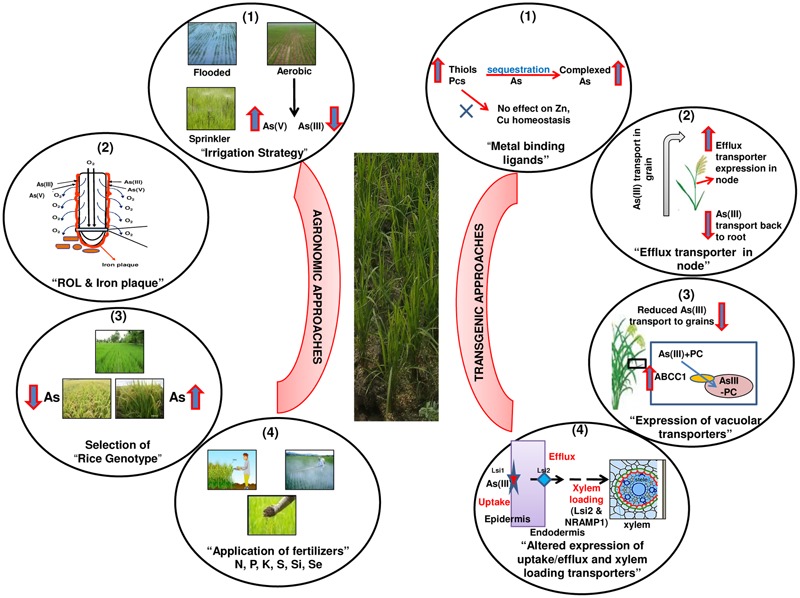
A hypothetical “Integrated Optimum Approach” to safeguard rice plants from arsenic toxicity and to achieve desirable reductions in arsenic in grains without affecting uptake, transport and homeostasis of essential nutrient elements. The Integrated approach means application of more than one approach in conjunction, while Optimum indicates achieving optimum concentrations of essential elements while targeting arsenic reduction in grains. Agronomic approaches: (1) Appropriate selection of irrigation method for maintaining proper soil moisture that favors AsV over AsIII (2) Properties of radial oxygen loss and iron plaque formation to be considered for the selection of best rice genotype having (3) low arsenic accumulation in grain and having high yields and (4) Application of appropriate amount of fertilizers to enrich the plants with nutrients and decrease As availability. Transgenic approaches: (1) Changes in expression of arsenic complexing ligands [glutathione (GSH), phytochelatins (PCs),] up to a level that increases As complexation but does not influence essential metal (Zn, Cu) homeostasis, (2) Expression of efflux transporters in upper nodes to facilitate the transport of As back toward roots, (3) Regulated expression of vacuolar transporters to aid in detoxification of As by sequestration of complexed As in vacuoles, and (4) Modulation of transporters for uptake, efflux and xylem loading to reduce As uptake as such and/or to concentrate As in roots.

Hence, there are a few potential strategies available that can be possibly applied to achieve the target of decreasing As in rice grains. However, contrasting responses and pros and cons of these strategies demand designing an “Integrated Optimum Approach” in future (**Figure 5**). Such a strategy would rely on the use of more than one approach (agronomic / transgenic) to be used in conjunction (Integrated) to yield high quality grains having safe and optimum levels of various elements (Optimum). This demands research in comprehensive manner in future to safeguard rice and humans from the As threat.

## Author Contributions

SS and RDT conceptualized the review. SA, RC, and SS wrote the review. SS and RDT did final editing of the MS.

## Conflict of Interest Statement

The authors declare that the research was conducted in the absence of any commercial or financial relationships that could be construed as a potential conflict of interest.
